# Ecosystem CO_2_ release driven by wind occurs in drylands at global scale

**DOI:** 10.1111/gcb.16277

**Published:** 2022-06-21

**Authors:** María Rosario Moya, Ana López‐Ballesteros, Enrique P. Sánchez‐Cañete, Penélope Serrano‐Ortiz, Cecilio Oyonarte, Francisco Domingo, Andrew S. Kowalski

**Affiliations:** ^1^ Experimental Station of Arid Zones (EEZA‐CSIC), Desertification and Geoecology Almería Spain; ^2^ Basque Centre for Climate Change (BC3) Scientific Campus of the University of the Basque Country Leioa Spain; ^3^ Department of Agricultural and Forest Systems and the Environment Agrifood Research and Technology Centre of Aragon (CITA) Zaragoza Spain; ^4^ Applied Physics University of Granada (UGR) Granada Spain; ^5^ Inter‐University Institute for Earth System Research (IISTA‐CEAMA) Granada Spain; ^6^ Ecology University of Granada (UGR) Granada Spain; ^7^ Agronomy University of Almeria (UAL) Almería Spain; ^8^ Andalusian Center for the Assessment and Monitoring of Global Change (CAESCG) Almería Spain

**Keywords:** drylands, eddy covariance, FLUXNET, global carbon cycle, ventilation

## Abstract

Subterranean ventilation is a non‐diffusive transport process that provokes the abrupt transfer of CO_2_‐rich air (previously stored) through water‐free soil pores and cracks from the vadose zone to the atmosphere, under high‐turbulence conditions. In dryland ecosystems, whose biological carbon exchanges are poorly characterized, it can strongly determine eddy‐covariance CO_2_ fluxes that are used to validate remote sensing products and constrain models of gross primary productivity. Although subterranean ventilation episodes (VE) may occur in arid and semi‐arid regions, which are unsung players in the global carbon cycle, little research has focused on the role of VE CO_2_ emissions in land–atmosphere CO_2_ exchange. This study shows clear empirical evidence of globally occurring VE. To identify VE, we used in situ quality‐controlled eddy‐covariance open data of carbon fluxes and ancillary variables from 145 sites in different open land covers (grassland, cropland, shrubland, savanna, and barren) across the globe. We selected the analyzed database from the FLUXNET2015, AmeriFlux, OzFlux, and AsiaFlux networks. To standardize the analysis, we designed an algorithm to detect CO_2_ emissions produced by VE at all sites considered in this study. Its main requirement is the presence of considerable and non‐spurious correlation between the friction velocity (i.e., turbulence) and CO_2_ emissions. Of the sites analyzed, 34% exhibited the occurrence of VE. This vented CO_2_ emerged mainly from arid ecosystems (84%) and sites with hot and dry periods. Despite some limitations in data availability, this research demonstrates that VE‐driven CO_2_ emissions occur globally. Future research should seek a better understanding of its drivers and the improvement of partitioning models, to reduce uncertainties in estimated biological CO_2_ exchanges and infer their contribution to the global net ecosystem carbon balance.

## Graphical abstract

Subterranean ventilation (VE) is a non‐diffusive transport process that provokes the abrupt transfer of CO_2_‐rich air through water‐free soil pores and cracks from the vadose zone to the atmosphere, under high‐turbulence conditions. This study shows clear empirical evidence of globally occurring VE. To identify VE, we designed an algorithm to detect CO_2_ emissions produced by VE at 145 open ecosystems selected from main eddy covariance networks. Of the sites analyzed, 34 % exhibited the occurrence of VE. This vented CO_2_ emerged mainly from arid ecosystems (84 %) and sites with hot and dry periods, which constitute the largest biome in the world.

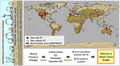

## 1 INTRODUCTION

The eddy‐covariance (EC) technique is a powerful tool (Aubinet et al., 2012; Baldocchi, 2014) that enables the continuous non‐destructive monitoring of mass, energy and momentum exchange between the land and the atmosphere (Baldocchi et al., 2001). Its use helps the scientific community to reach a better understanding of how terrestrial ecosystems interact with the Earth's climate system, an outstanding scientific challenge in the global change context (Reich, 2010; Solomon et al., 2007). With a growing number of EC towers worldwide, a set of regional (e.g., Ameriflux, CarboEurope, AsiaFluz, OzFlux; Aubinet et al., 1999; Isaac et al., 2016; Novick et al., 2018; Reich, 2010; Yu et al., 2006) and global networks (FLUXNET; Pastorello et al., 2020) of EC flux measurement stations has been established. However, although arid and semiarid lands comprise approximately 40% of the global terrestrial surface (Reynolds et al., 2007) and play an important role in the global carbon (C) cycle (Ahlström et al., 2015; Lal, 2004), observational gaps (Baldocchi, 2008; Bell et al., 2012; Schimel et al., 2015; Xiao et al., 2008) have hampered a full understanding of their functional behavior.

As a result, the interpretation of net CO_2_ fluxes derived from EC measurements has been constrained by available data and research, which has been extensively developed in temperate ecosystems where CO_2_ fluxes have commonly been attributed purely biological processes (i.e., gross primary production, GPP, and ecosystem respiration, R_eco_) that occur within the ecosystem boundaries (Chapin et al., 2006; i.e., from the upper soil layers to the close‐to‐surface atmosphere). However, in water‐limited ecosystems, there is evidence of other processes, besides biological processes GPP and R_eco_, that can significantly contribute to net CO_2_ flux observed by the EC technique, at least on short time scales (Emmerich, 2003; Mielnick et al., 2005; Serrano‐Ortiz et al., 2010). These include photodegradation (Rutledge et al., 2010), geochemical weathering (Hamerlynck et al., 2013), and subterranean ventilation (Kowalski et al., 2008). Neglecting these processes when partitioning net CO_2_ fluxes can result in biased estimates of GPP and R_eco_ fluxes (Ferlan et al., 2011; Inglima et al., 2009; Mielnick et al., 2005; Were et al., 2010; Xie et al., 2009), which are extensively utilized to calibrate models and satellite products (Jones & Cox, 2005; Ma et al., 2015; Verma et al., 2014) to assess current and future coupling between the terrestrial biosphere and the climate system (Naidu & Bagchi, 2021; Poulter et al., 2014). Here we pay special attention to subterranean ventilation, whose relevance is suggested to be notable compared to other contributing processes, particularly at those ecosystems where water scarcity over the drought season inhibits biological processes (López‐Ballesteros et al., 2017; Pérez‐Priego et al., 2013).

Subterranean ventilation is a non‐diffusive transport process that provokes the abrupt transfer of CO_2_‐rich air from the vadose zone to the atmosphere under drought and high‐turbulence conditions (Kowalski et al., 2008). Recent studies developed in drylands have pointed out the importance of the vadose zone as a significant temporary CO_2_ pool, given its capacity to store vast amounts of gaseous CO_2_ in interconnected cracks, pores, and cavities belowground where volumetric CO_2_ fractions can rise to two orders of magnitude greater than in the atmosphere (Benavente et al., 2010; Faimon et al., 2020; Fernandez‐Cortes et al., 2015; Sánchez‐Cañete et al., 2016). Regardless of its originating process, the enhanced density of CO_2_‐rich air (Kowalski & Sánchez‐Cañete, 2010)—due to its large molecular mass—may enable it to descend from shallow soil layers, where most root and microbial respiration takes place (Buchmann, 2000; Coleman & Crossley, 2004, down to the water table level, creating a concentration gradient that increases with depth. In order for subterranean ventilation episodes (VE) to happen, several conditions are required. First, low values of soil water content must be reached to allow gas flow across the porous medium between the vadose zone and the atmosphere (Cuezva et al., 2011; Risk et al., 2002; Roland et al., 2013; Serrano‐Ortiz et al., 2010). This condition commonly occurs during daytime and especially during the dry season coinciding with the dormancy period, when water scarcity and high temperatures constrain biological activity (i.e., CO_2_ emissions are likely inconsistent with simultaneous variations of organic carbon pools; Huxman et al., 2004). Conversely, during nighttime, atmospheric stability, water deposition, and vapor adsorption near the soil surface may limit this belowground and aboveground air interconnection. Second, high atmospheric turbulence conditions are indispensable to penetrate the vadose zone and produce the abrupt release of CO₂ from the vadose zone to the atmosphere via non‐diffusive transport (Kowalski et al., 2008; Subke et al., 2003; Takle et al., 2004). For this reason, the wind velocity (or friction velocity) is considered the main trigger of this phenomena (Flechard et al., 2007; Maier et al., 2010; Redeker et al., 2015). Third, the air located in the vadose zone must be significantly CO_2_ rich (a high storage term is needed) to make VE detectable for the EC system. While the first and second conditions (i.e., low soil water content, and high turbulence) can be considered as predisposing factors enabling VE to occur, the third condition (i.e., vadose zone CO_2_ concentration) directly affects the magnitude of VE‐driven CO_2_ emissions (Lang et al., 2017; Sanchez‐Cañete et al., 2011).

Recent research has demonstrated that subterranean ventilation can temporarily dominate land–atmosphere CO_2_ exchange in some Mediterranean ecosystems (Faimon et al., 2020; López‐Ballesteros et al., 2017; Pérez‐Priego et al., 2013; Sanchez‐Cañete et al., 2011). These studies were mainly developed in Southwestern Europe and utilized specific experimental setups designed to carefully monitor the influencing factors mentioned above. However, the relevance of subterranean ventilation at global scale is still unknown. The general aim of this study is to test whether VE happen globally by analyzing flux data provided by FLUXNET and other related regional networks. Our main hypothesis is that VE occur in water‐limited ecosystems worldwide, and particularly during the dry season. To test our hypothesis, we designed an algorithm, based on previous scientific evidence, to systematically detect VE in the 145 open ecosystems selected for this study. Therefore, our specific objectives are as follows: (1) to detect daytime VE‐driven CO_2_ emissions in different ecosystems distributed around the world and (2) to investigate factors influencing VE.

## 2 MATERIALS AND METHODS

### 2.1 Database selection

To identify VE we used in situ, quality‐controlled eddy‐covariance observations of carbon dioxide fluxes and ancillary data from FLUXNET and main regional EC networks that are available as open data to the science community. The datasets used in this study include the FLUXNET2015 (https://fluxnet.fluxdata.org), the AmeriFlux (http://ameriflux.lbl.gov), the OzFlux (http://www.ozflux.org.au), and the AsiaFlux (http://www.asiaflux.net) databases.

Flux sites selection was limited to non‐forested open ecosystems (Bond, 2019). Thus, our analysis was limited to the following land covers based on the IGBP classification (https://fluxnet.org/data/badm‐data‐templates/igbp‐classification/): grasslands (GRA), savannas (SAV), woody savannas (WSA), open shrublands (OSH), closed shrublands (CSH), barrens or sparsely vegetates (BSV), and croplands (CRO). This selection yielded 588 site‐years of EC data measured at 145 sites around the world. The sites selected were also categorized by climate using Köppen classification (KGCC; Rubel & Kottek, 2010), and the world dryland areas dataset (UNEP‐WCMC; Sorensen, 2007).

The basic instrumentation of EC technique consists of a sonic anemometer (to measure 3D wind velocity and virtual temperature at high frequency), an infrared gas analyzer (to measure CO_2_ and water vapor densities at high frequency), and complementary sensors to measure other meteorological ancillary variables at the flux site (e.g., air temperature, relative humidity, net radiation, precipitation). The flux and meteorological half‐hourly data used in this analysis corresponded to quality‐controlled data directly measured (i.e., minimum processing level). Therefore, we excluded post‐processed gap‐filled data (Papale & Valentini, 2003; Reichstein et al., 2005) from our analysis.

### 2.2 Data processing method

We designed a novel algorithm to automatically detect VE‐driven CO_2_ emissions among the analyzed flux data. Our algorithm aims at detecting VE over periods when subterranean ventilation may prevail over other processes. The algorithm was designed based on previous research reflected in the following assumptions: (1) atmospheric turbulence must be sufficient to pump CO_2_‐rich air from the vadose zone to the atmosphere; (2) no nocturnal VE happens due to soil re‐humidification; and (3) the required variability in air temperature and soil moisture must be insufficient to explain variations of net CO_2_ emissions (i.e., incompatible with an ecophysiological interpretation of fluxes, such as the Birch effect at the end of the dry season).

A temporal window is needed to statistically discriminate subterranean CO_2_ release due to changes in air temperature (e.g., with the passage of high‐ and low‐pressure systems and fronts) or soil moisture. Although VE is an abrupt process (the smallest time window over which a VE could be identified is <1 s; Kimball & Lemon, 1970; Massman et al., 1997; Mohr et al., 2016; Takle et al., 2004), considering previous research (Sanchez‐Cañete et al., 2011), a period of 5 days with sustained high turbulence, similar mean air temperature and dry conditions were considered as unequivocal to detect VE. Thus, we applied the algorithm to 5‐day intervals over 1 year for each selected site.

In accordance with the above assumptions, we filtered these data according to the following criteria: (1) only daytime (shortwave radiation incoming [SW_IN] or photosynthetic photon flux density incoming [PPFD_IN] > 50 W m^−2^), (2) positive fluxes (net ecosystem exchange [NEE] or net carbon dioxide flux [F_c_] > 0 μmolCO_2_ m^−2^ s^−1^), (3) maximum mean air temperature (TA) absolute difference between days 1 and 5 of 3°C, (4) maximum mean soil water content (SWC) absolute difference between days 1 and 5 of 1%, (5) null precipitation [P > 0.00001], and (6) high‐turbulence conditions (u_*_ > 0.2 m s^−1^) data were used. Furthermore, to reduce the potential effect of data availability on the number of VE detected our algorithm, only 5‐day intervals with a minimum number of data (N > 40) and CO_2_ maximum quality (flag qc = 0) were used. Finally, we gathered an indicator of data availability for each site and year that were analyzed (see Table S1).

We computed Partial Spearman correlation coefficients over each 5‐day period filtered to eliminate spurious correlation effects between F_c_ (μmolCO_2_ m^−2^ s^−1^) and ancillary data. Ancillary variables considered in partial correlation were as follows: air temperature, vapor pressure deficit, soil temperature, atmospheric pressure, soil water content, incoming photosynthetic photon flux density, incoming shortwave radiation and friction velocity. Only 5‐day intervals with partial Spearman correlation coefficients between F_c_ and u_*_ above 0.2 and *p* < .05 (support the evidence of the alternative hypothesis, i.e., non‐zero partial correlation, being significantly different from the null hypothesis, i.e., zero partial correlation) were considered as VE. The software Matlab was used for statistical analyses (Matlab R2017a).

To balance the different dataset duration among analyzed sites as well as to simplify our analysis, results shown in this study correspond to 1 year of data per site. We have considered the assumption that experimental sites where VE were not detected after analyzing 4 years of data are not VE predisposed sites (in accordance with our algorithm design). Thus, the algorithm was performed to the available datasets under the next conditions: (1) for site‐specific databases lasting from 1 to 4 years, the algorithm was applied to the whole database; (2) for site‐specific databases lasting from 5 to 6 years, the algorithm was applied to the first four consecutive years; and (3) for site‐specific databases lasting more than 6 years, the algorithm was applied to the first four non‐consecutive years. In those sites where VE‐driven CO_2_ emissions were detected over several years, the year finally selected corresponded to the one with more VE detected. The list of FLUXNET and regional EC networks sites used in this study with respective basic information appears in the supporting information (Tables [Link], [Link]).

## 3 RESULTS

**FIGURE 1:**
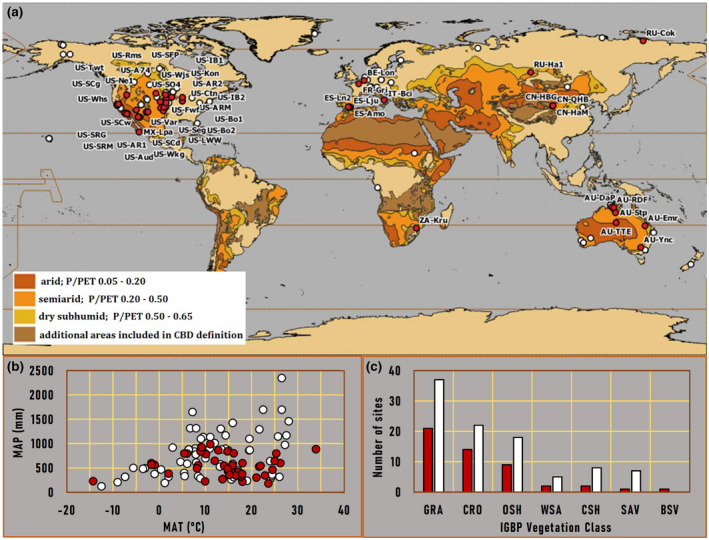
(a) World map distribution of FLUXNET2015, AmeriFlux, OzFlux, and AsiaFlux sites analyzed in this study with distinction of experimental sites with (red marker) and without (white marker) VE‐driven CO_2_ emissions detected by our algorithm. Map colors indicate world dryland areas dataset classification (UNEP‐WCMC; Sorensen, 2007) according to dryness index PET/P (potential evapotranspiration/precipitation). Codes of sites with VE‐driven CO_2_ emissions have been included; (b) distribution of analyzed sites with (red) and without (white) VE emissions along mean annual temperature (MAT) and mean annual precipitation (MAP) gradients; (c) number of analyzed sites with (red) and without (white) VE emissions detected by IGBP land cover classification (grassland, GRA; savanna, SAV; woody savanna, WSA; open shrubland, OSH; closed shrubland, CSH; barren or sparsely vegetated, BSV; and cropland, CRO). CBD is the acronym for convention on biological diversity.

Of the 145 sites analyzed in our study, we found that 34% (50 sites) presented VE‐driven CO_2_ emissions according to the established criteria (Figure 1). Of these, 84% (42 of 50) are located in drylands, in accordance with the United Nations Convention to Combat Desertification (UNCCD) definition of Drylands (UNEP‐WCMC; Sorensen, 2007). These VE‐driven CO_2_ emissions were frequent among the different drylands' ecosystem types, occurring in half (3 of the total 6) of arid drylands, 56% (24/43) of semiarid drylands, 57% (13/23) of dry subhumid drylands, and 64% (18/28) of additional dryland areas.

Australia and the western USA were the main regions where these anomalous CO_2_ emissions occurred. However, the distribution of EC stations conditioned this proportion. From the ecosystems analyzed in Europe, 30% of them (6/20) gave positive results with half of these being located in SE Spain. In Asia, 42% (5/12) of the experimental sites analyzed showed positive results with half of these located in the Tibetan Plateau. In the United States and Mexico, 34% (32/93) of the experimental sites analyzed gave positive results. These places are located in the lower part of the United States, in the middle and on the west coast. Finally, in Australia, 32% (6/19) of the experimental sites analyzed gave positive results. These places are located in the middle and east part of the country. Unluckily due to the lack of observations in South America and Africa, there are no positive episodes detected in South America and only one of three ecosystems analyzed in South Africa (33%) exhibited VE.

The mean annual precipitation was below 1000 mm at all the experimental sites with VE detected (Figure 1B). The mean annual temperature ranges between −1 and 26°C, although two ecosystems (Ru‐Cok and US‐A74 with −14.3°C and 33.9°C, respectively) far exceeded this range. The ecosystem types with more VE corresponded to grasslands, croplands, and open shrublands, in that order. Of the remaining ecosystems analyzed, only one (in savannas and barrens or sparsely vegetates sites) and two places (in woody savannas and closed shrublands sites) showed VE. The supporting information (S1, S2, and S3) gives more details for each experimental site.

**FIGURE 2:**
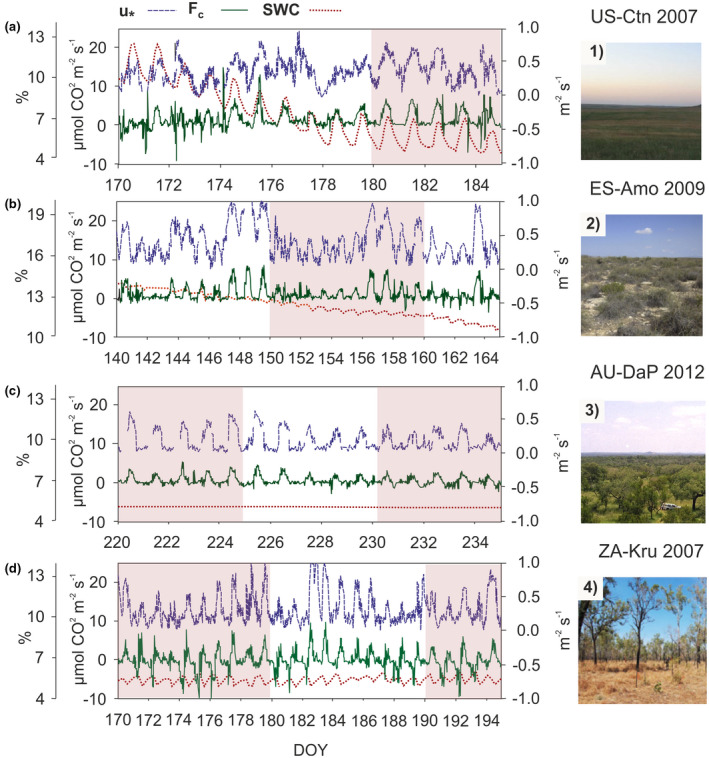
Average half‐hour time series of friction velocity (u_*_ in blue; m s^−1^), ecosystem‐atmosphere net CO_2_ fluxes (F_c_ in green; μmolCO_2_ m^−2^ s^−1^), and soil water content (SWC in red; %) in: (a.1) US‐Ctn (cottonwood, grassland, United States, 2007, AmeriFlux); (b.2) ES‐Amo (Amoladeras, open shrubland, Spain, 2009, FLUXNET); (c.3) AU‐DaP (Daly river savanna, grassland, Australia, 2012, FLUXNET); (d.4) ZA‐Kru (Skukuza, savanna, South Africa, 2007, FLUXNET) experimental sites. A negative F_c_ denotes carbon uptake by the ecosystem, and a positive F_c_ denotes emissions to the atmosphere. Red shaded areas in each figure denote the 5‐day periods for which our algorithm detected VE. Photos from https://fluxnet.org/

Figure 2 shows the selection of four experimental sites with VE detected by our algorithm, chosen to exemplify driving processes and patterns. US‐Ctn is a grassland located in United States classified as cold arid steppe (BSk); ZA‐Kru is a savanna located in South Africa classified as hot arid steppe (BSh); ES‐Amo is an open shrubland located in Spain and classified as a cold arid steppe (BSk); and AU‐Dap is a grassland located in Australia and classified as equatorial with dry winter (Aw). Although these are ecosystems located in faraway regions, the ecosystem's dynamic is very similar among them (Figure 2). These sites revealed a seasonal variability with two contrasting CO_2_ exchange behaviors during two predominant periods: the growing and dry season. During growth periods, these sites acted as net daily carbon sinks (not shown). However, during dry periods (when plants are dormant), these sites acted as net carbon sources (Figure 2). Similar daily patterns were noticeable for SWC, F_c_ and u_*_ variables. The positive relationship between the intensity of the atmospheric turbulence (u_*_) and the occurrence and magnitude of the CO_2_ emissions (positive F_c_) is consistent for all these sites, especially when SWC was very low. In fact, VE occurred in US‐Ctn and AU‐DaP sites when soil moisture values reached their 20th percentile (computed on an annual basis). Similarly, in ES‐Amo and ZA‐Kru, VE coincided with soil moisture values close to their 30th percentiles. This SWC threshold was also evaluated in the other 46 experimental sites with VE and was fulfilled in sites classified as “dry subhumid,” “semiarid,” and “arid” in the dryland type classification (UNEP‐WCMC). To point this assumption, the US‐Ctn site (Figure 2a), which showed a progressive decrease in SWC values of one order of magnitude over a 15‐day time window, has been graphed during the “pre‐emission” and the beginning of the “emission” period. When the SWC reached its 30th percentile in US‐Ctn, our filter detects the first 5‐day periods of emissions (DOY 180–184). In contrast, ES‐Amo, ZA‐Kru and AU‐DaP sites (Figure 2b–d), where SWC values were less variable, have been graphed during the “emission” period with more VE detected on previous days (not shown in Figure 2). Although there are a few days with turbulent conditions (high u_*_) during night (DOY 174–179 in US‐Ctn; Figure 2a), these do not coincide with nighttime CO_2_ emissions. For the selected year, the length of the CO_2_ release periods (with positive F_c_) was 258 days in ES‐Amo (DOY 97–355), 47 days in US‐Ctn (DOY 175–222), 149 days in AU‐DaP (DOY 158–307), and 249 days in ZA‐Kru (DOY 54–303).

**FIGURE 3:**
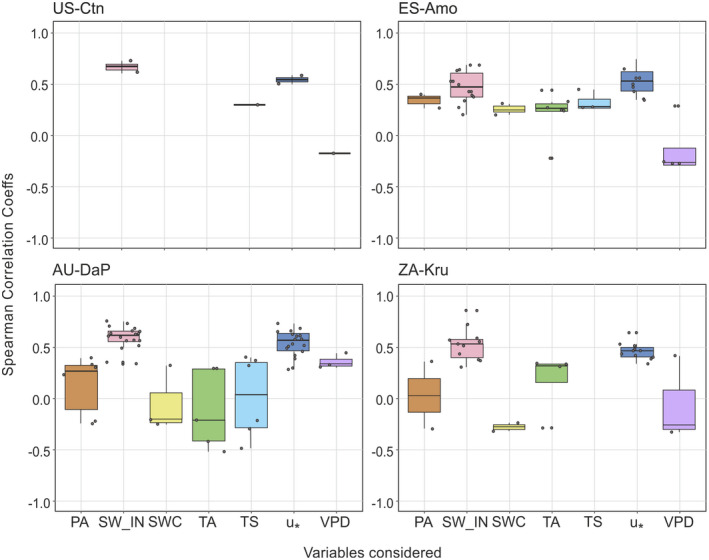
Box‐and‐whisker plots of spearman coefficients obtained from partial correlation between CO_2_ fluxes (F_c_) and friction velocity (u_*_), air temperature (TA), vapor pressure deficit (VPD), atmospheric pressure (PA), soil temperature (TS), soil water content (SWC) or shortwave radiation incoming (SW_IN) for the 5‐day periods when VE‐driven CO_2_ emissions were detected at ZA‐Kru, ES‐Amo, US‐Ctn, and AU‐DaP sites. Each Spearman correlation coefficient obtained was not influenced by the indirect effect of the remaining variables considered in the analysis. Each data shown have an individual associated *p*‐value <.05 (i.e., partial correlation coefficient different from zero).

VE detected in the four experimental sites selected were directly correlated with u_*_ and the shortwave incoming radiation SW_IN (Figure 3). The value of the F_c_‐u_*_ partial correlation or the F_c_‐SW_IN partial correlation varies among the different VE detected; however, the median was similar among ecosystems (between 0.44 and 0.55 the F_c_‐u_*_ correlation and 0.45–0.67 the F_c_‐SW_IN correlation). During these periods, there were also positive and negative correlations with other variables measured in the ecosystems: air temperature (TA), vapor pressure deficit (VPD), atmospheric pressure (PA), soil temperature (TS), or/and soil water content (SWC). However, these correlations were more heterogeneous among ecosystems, weaker, and not always significant.

**FIGURE 4:**
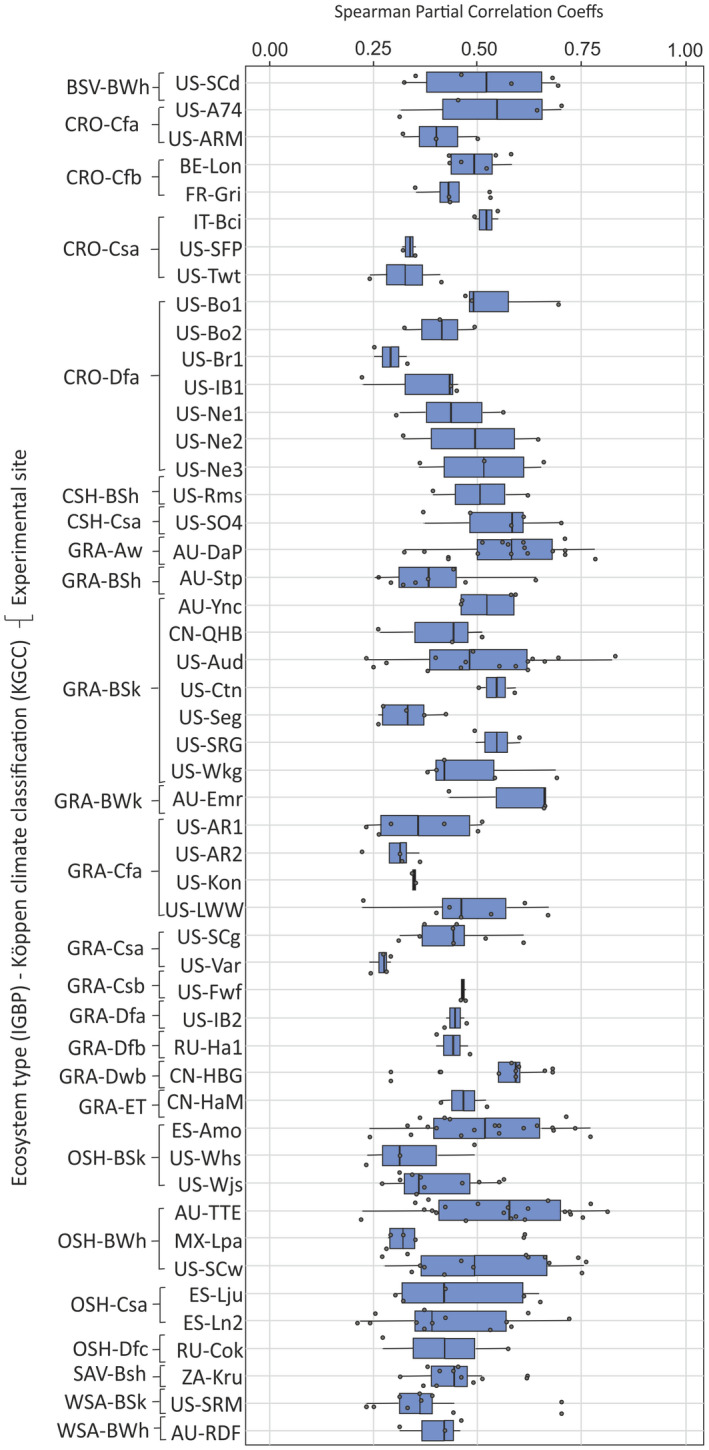
Box‐and‐whisker plots of Spearman coefficients obtained from partial correlation between CO_2_ fluxes (F_c_) and friction velocity (u_*_) for all the flux sites where 5‐day VE were detected by our algorithm. Each Spearman correlation coefficient obtained was not influenced by the indirect effect of the rest of covariables considered: Air temperature, vapor pressure deficit, atmospheric pressure, soil temperature, soil water content, and shortwave radiation incoming; for the 5‐day periods when VE‐driven CO_2_ emissions were detected at each experimental site. Sites cluster by ecosystem type [land cover (IGBP)] and Köppen climate classification (KGCC). Each data shown have an individual associated *p*‐value <.05 (i.e., partial correlation coefficient different from zero).

In the 50 sites with VE‐driven CO_2_ emissions, our algorithm detected 290 VE according to the established criteria. The number of VE detected were positively related to the aridity degree represented via the dryland type classification (UNEP‐WCMC) and negatively related to the normalized SWC. The Spearman partial correlation coefficients obtained between F_c_ and u_*_ ranged from 0.2 to 0.8 with a median value around 0.5 (Figure 4). The climatic zones with the highest Spearman correlation coefficients were BWh (hot desert climate), BWk (cold desert climate), and Aw (equatorial savannah with dry winter), while BSk (cold steppe climate), CSa (warm temperate climate with hot and dry summer), Cfa (warm temperate climate, fully humid with hot summer), and Dfa (Snow climate, fully humid with hot summer) showed the lowest Spearman correlation coefficients. Regarding VE occurrence frequency, BSk (28%) and BWh (18%) are the climatic zones with more VE detected, while Csb (warm temperate climate with warm and dry summer; 0.7%) and Dfb (snow climate, fully humid with warm summer; 1.3%) are the climatic zones with less VE detected.

By region, the 50% of the Australian sites analyzed showed the highest coefficient median (AU‐Emr [GRA‐BWk], CN‐HBG [GRA‐DWb], AU‐DaP [GRA‐Aw], US‐SO4 [CSH‐Csa], and AU‐TTE [OSH‐BWh]). While the sites with the lowest coefficient median are located in United States and Mexico (US‐Whs [OSH‐BSk], MX‐Lpa [OSH‐BWh], US‐Var [GRA‐Csa], US‐AR2 [GRA‐Cfa], US‐Twt [CRO‐Csa], US‐SPF [CRO‐Csa], and US‐BR1 [CRO‐Dfa]). By land cover, grasslands (GRA), and open shrublands (OSH) are the ecosystem types with the highest and lowest partial Spearman correlation coefficients, respectively. The Spearman correlation coefficients obtained between F_c_ and u_*_ for each experimental site analyzed are found in Table S1.

## 4 DISCUSSION

Our results suggest that CO_2_ emissions driven by subterranean ventilation occur globally. After analyzing 145 open ecosystems (Bond, 2019) selected from main EC networks, our algorithm, designed upon previous scientific evidence, detected ventilation episodes (VE) in 34% of them (Figure 1a, and Tables S1 and S2). Our main hypothesis was corroborated as 84% of the analyzed sites corresponded to open dryland ecosystems based on the IGBP classification and the UNCCD definition of Drylands (Sorensen, 2007). The detection of VE was quite balanced among the different kinds of drylands presented (50% in arid, 56% in semiarid, 57% in dry subhumid, and 64% in the additional dryland areas). During these VE, our results show that the relationship between net CO_2_ fluxes and air and soil temperature, vapor pressure deficit, atmospheric pressure, and soil water content were more heterogeneous among ecosystems, weaker, and not always significant, compared to incoming shortwave radiation and atmospheric turbulence (Figure 3). These results could be explained by the variability in the predisposing factors among VE occurred in a same site and suggest the predominance of subterranean ventilation over biological processes (i.e., GPP or R_eco_) during drier periods.

The occurrence and magnitude of the VE‐driven CO_2_ release from the vadose zone to the atmosphere were directly related with the intensity of the atmospheric turbulence (u_*_ value; Figures 2, 3, 4). The Spearman coefficients obtained from partial correlation between F_c_ and u_*_ found among the climate‐IGPB categories ranged from 0.2 and 0.8 with a median value around 0.5 (Figure 4). These results are in accordance with the assumption stating that eddies promoted by high atmospheric turbulence conditions can penetrate the soil matrix and produce an abrupt emission of CO_2_‐rich air from the vadose zone to the atmosphere via non‐diffusive transport (Kowalski et al., 2008; Subke et al., 2003; Takle et al., 2004). The variability of the coefficients for a site could then be explained by the different CO_2_ concentrations in the vadose zone for each VE (no data available) and variability in the predisposing factors among VE occurred in the same site. Just as nocturnal VE were assumed as non‐existent in our analysis, in the four experimental sites selection shown in Figure 2, during nights with turbulent conditions (DOY 174–179 in US‐Ctn) there were not nighttime CO_2_ emissions. As pointed out by other authors, at night water deposition and vapor adsorption near the soil surface may inhibit ventilation (Agam & Berliner, 2006; Cuezva et al., 2011; Kosmas et al., 2001; Verhoef et al., 2006).

Most VE occurred during dry conditions, with SWC values below the 30th percentile of site‐specific annual soil moisture in sites classified as “dry subhumid,” “semiarid,” and “arid” in the dryland type classification (UNEP‐WCMC). Such behavior can be clearly observed in US‐Ctn site (Figure 2a), where the first 5‐day period of emissions (DOY 180–184) was detected when the SWC decreased down its 30th percentile during daytime and over the dry season. Due to the lack of water inputs, the interconnectivity between vadose zone and the atmosphere can allow for an effective gas transport across the land surface (Cuezva et al., 2011; Risk et al., 2002; Roland et al., 2013; Sanchez‐Cañete et al., 2011). This dryness condition can correspond to an inherent feature of the ecosystem (i.e., US‐Ctn, AU‐DaP or ES‐Amo) or to a climate extreme, such as a drought event (i.e., ZA‐Kru). It is also remarkable how the mean annual precipitation is a limiting factor, which was below 1000 mm in all the experimental sites with VE detected (Figure 1b).

The shortwave incoming radiation (SW_IN) also constitutes an important meteorological variable involved in the VE‐driven CO_2_ emissions detected (Figure 3) probably because solar heating is an important mechanism that triggers turbulence (López‐Ballesteros et al., 2017; Stull, 1988). In relation with the VPD variable, López‐Ballesteros et al. (2017) found that VE‐driven CO_2_ emissions coincided with high turbulence and high VPD conditions; however, our results do not clearly show such positive VPD‐VE relationship.

In relation with the F_c_‐pressure correlation, several processes would be involved. Pressure pumping (Massman et al., 1997; Mohr et al., 2016; Roland et al., 2015), pressure tides (Clements & Wilkening, 1974; Kimball & Lemon, 1970; le Blancq, 2011), and subterranean ventilation are non‐diffusive transport processes affected by atmospheric pressure changes at different temporal scales. Enabling conditions for all of these processes are a high subsoil–atmosphere interconnectivity (López‐Ballesteros, Oyonarte, et al., 2018; Pérez‐Priego et al., 2013; Sánchez Cañete, Kowalski, et al., 2013; Sánchez Cañete, Serrano Ortiz, et al., 2013), which, in turn, relies on low SWC values and high atmospheric turbulence, as these are indispensable to allow for the CO_2_‐rich air transfer from the vadose zone to the atmosphere. For this reason, it is challenging to distinguish among pressure pumping, pressure tides, and subterranean ventilation. Some authors have shown a positive relationship between pressure pumping and ventilation (Mohr et al., 2016; Nachshon et al., 2012; Redeker et al., 2015), based on the correlation shown between pressure changes and wind perturbations at high frequencies (Maier et al., 2010; Redeker et al., 2015; Takle et al., 2004). However, other authors (Moya et al., 2019; Sánchez Cañete, Kowalski, et al., 2013) found a temporal and spatial decoupling between VE and pressure tides. Previous research states that pressure tides were produced in the sub‐surface at low frequencies by large‐scale atmospheric dynamics (Elberling et al., 1998; Guoqing, 2005; Lindzen, 1979), while VE is produced at high frequencies and limited to the shallower horizon (Redeker et al., 2015; Takle et al., 2004). Regarding subsoil–air interconnectivity, we did not find any relation between site‐specific soil type and occurrence of VE (data not shown); however, low SWC values are related to a minimum gas permeability of the soil matrix needed to enable VE (Pérez‐Priego et al., 2013; Sánchez‐Cañete et al., 2016).

Due to water dependency, flux measurements acquired in drylands reveal two contrasting CO_2_ exchange behaviors during two predominant periods: the growing and dry seasons (López‐Ballesteros et al., 2017; Pérez‐Priego et al., 2013; Serrano Ortiz et al., 2014). During growing periods, sufficient soil moisture enables autotrophic and heterotrophic biological activity and inhibits VE by lowering subsoil–atmosphere interconnectivity. However, during dry periods, the low soil moisture reduces biological processes involved in the absorption and production (and consequent emission) of CO_2_ to a secondary role, allowing VE to become more relevant. Therefore, the high inter‐annual variability of F_c_ in ES‐Amo and ZA‐Kru could be a consequence of the frequency and magnitude of VE. ES‐Amo acted as an annual net source of CO_2_ ranging from +163 g C m^−2^ yr^−1^ to +324 g C m^−2^ yr^−1^ over 2009–2015 (López‐Ballesteros et al., 2017), whereas ZA‐Kru varied from −138 to +155 g C m^−2^ yr^−1^ over 2000–2005 (Archibald et al., 2009). Differences in the F_c_ balance among years depended on the length and severity of the dry season, which was determined by the magnitude and timing of precipitation events (Gilmanov et al., 2006; Luo et al., 2007; S. Ma et al., 2007; Meyers, 2001; Pereira et al., 2007). Future climate projections suggest an increase in long‐term droughts in the Mediterranean, west African, central Asian, and central American regions (Sheffield & Wood, 2008). Based on our results, this trend might be related to a higher occurrence of VE among these areas as minimum soil moisture is a prerequisite for VE to occur. However, it is not sure that drier drylands (in terms of drought duration and severity) will release more CO_2_ to the atmosphere via subterranean ventilation, as one of the main factors that presumably determine the magnitude (and occurrence) of this ventilative CO_2_ fluxes is the availability of CO_2_ in the air stored in the vadose zone to be ventilated (Lang et al., 2017; Sanchez‐Cañete et al., 2011).

Vadose zone can be considered as a spatial and temporal depot for CO_2_ coming from different processes (Bourges et al., 2001; Chapin et al., 2006; Serrano‐Ortiz et al., 2010). Depending on (1) the dimension and potential capacity of vadose zone subterranean CO_2_ storage, (2) the occurrence of VE, (3) the simultaneous occurrence of other sink (e.g., dissolved CO_2_ infiltration) and source mechanism (e.g., soil respiration), (4) changes in the drivers controlling the VE potential, and (5) changes in the degree of connection between the cavities and the aboveground system; the vadose zone net CO_2_ balance could be negative and the subterranean systems could be degassed. In this situation, even if predisposing factors (low values of soil water content and high atmospheric turbulence conditions) are present, the lack of subterranean CO_2_ available could disabled the occurrence of VE. Unfortunately, due to the absence of observations, we could not assess how vadose zone CO_2_ concentration varies and affects VE‐driven CO_2_ emissions among the analyzed sites.

In general, vadose zone CO_2_ may not necessarily be produced simultaneously and within the ecosystem where VE are detected. Based on previous research, three hypotheses could explain potential origins of the ventilated CO_2_.

The first hypothesis (H1) states that CO_2_ is produced in situ (i.e., within the ecosystem boundaries; Chapin et al., 2006) by microbial and root respiration, with delayed release to the atmosphere. In this regard, apparent respiratory quotient (ARQ) measurements performed in two semiarid ecosystems revealed that actual soil CO_2_ efflux only accounts for 1/3 and 1/4 of the total CO_2_ emissions derived from soil respiration (Angert et al., 2015; Sánchez‐Cañete et al., 2018). These studies demonstrated that not all CO_2_ produced by microbial and root respiration is immediately released to the atmosphere. Potential processes involved in this discrepancy are suggested to be aqueous phase partitioning (Kern, 1960), calcite dissolution reactions (Serrano‐Ortiz et al., 2010), gravitational percolation due to a higher density (Sánchez Cañete, Serrano Ortiz, et al., 2013), or CO_2_ dissolution in xylem water (Aubrey & Teskey, 2009). However, in the ES‐Amo site included in the present study, annual VE‐driven CO_2_ emissions measured systematically during six consecutive years were close to the estimated total soil organic carbon (SOC), suggesting that the origin of the ventilated CO_2_ could not be explained by previous in situ biological activity (López‐Ballesteros et al., 2017).

The second hypothesis (H2) states that CO_2_ can be produced ex situ (i.e., out of the ecosystem boundaries) by respiration but transported laterally in aqueous and gaseous CO_2_ forms. In this sense, Li et al. (2015) found in a closed arid basin that biological CO_2_ produced at higher altitudes were leached as dissolved inorganic carbon (DIC) and accumulated in the groundwater beneath the basin center.

Finally, the third hypothesis (H3) relates the CO_2_ production to non‐biological processes. With regard, some estimations of CO_2_ fluxes caused by photodegradation of senescent organic matter correspond to low CO_2_ effluxes of <0.2 μmol m^−2^ s^−1^ (Austin & Ballaré, 2010; Austin & Vivanco, 2006; Brandt et al., 2009; King et al., 2012; Rutledge et al., 2010). Likewise, short‐term estimates of geochemical weathering and calcite precipitation seemed to correspond to very low CO_2_ effluxes of ca. 0.05 μmol m^−2^ s^−1^ (Goddéris et al., 2006; Hamerlynck et al., 2013; Roland et al., 2013; Serrano‐Ortiz et al., 2010). Thus, these two processes are far from sufficient to explain the large magnitude of the daytime CO_2_ emissions detected by our algorithm (Figure 2 and Table S1). However, karstic areas deserve special attention given their great capacity to temporally store large amounts of gaseous CO_2_ belowground (Decarlo & Caylor, 2020; Emmerich, 2003; Serrano‐Ortiz et al., 2010) within their carbonate vadose zone, which usually contains an interconnected system of caves, macropores, and fissures. According to Faimon et al. (2020), these places can be considered as “breathing spots” that may be relevant in the carbon cycle at basin scale. Finally, another non‐biological process that might be involved in vadose zone CO_2_ production is related to deep‐seated CO_2_ seepage from porous reservoirs usually related to volcanic or hydrothermal activity (Burton et al., 2013; Chiodini et al., 2008; Lewicki et al., 2003; Mörner & Etiope, 2002; Rey et al., 2012). However, to know how each mentioned process interact with VE, we need further investigation at site level.

The EC databases used in this study presented some other limitations to provide more in‐depth information on the global VE relevance. These limitations include the following: (1) the presence of long‐term gaps in many databases, which made it difficult to calculate the contribution of VE at the annual scale; (2) the limited length of the time series (the majority of EC flux sites analyzed are usually limited to only 3–5 years of measurements) could be an issue (Schimel et al., 2015; Sulkava et al., 2011; Sundareshwar et al., 2007) to determine the effect of VE in the interannual variation of the ecosystem C balance; (3) the heterogeneity among databases (absence of some variables collected and differences in data quality) and the lack of information and/or parameter misspecification in the metadata made it difficult to establish relations between VE and other variables across the different types of ecosystems; and (4) the spatial representativeness in the EC databases (López‐ballesteros, Beck, et al., 2018; Schimel et al., 2015; Sulkava et al., 2011; Sundareshwar et al., 2007) was not sufficient to capture the natural variability of climatological and biological conditions in some geographical regions. FLUXNET and regional network sites are mainly located in ecosystems with annual temperatures between 5 and 17°C, annual rainfall between 600 and 1250 mm, and latitudes above 30°N (Vargas et al., 2013). Furthermore, longer datasets are limited to temperate and boreal ecosystems (Chu et al., 2017; Pastorello et al., 2017, 2020). Thus, regions more vulnerable to environmental or climate change are still underrepresented (Bell et al., 2012). Many of these regions are drylands or prone to desertification (Emmerich, 2003; Eswaran et al., 2000; Huenneke et al., 2002; Schlesinger, 1990), which makes VE potentially relevant now and even more so in the future (Ahlström et al., 2015). Furthermore, the lack of CO_2_ concentration measurements within the vadose zone limited our analyses, as this is the main factor driving the magnitude of VE‐driven CO_2_ emissions (Sanchez‐Cañete et al., 2011; Serrano‐Ortiz et al., 2010).

These issues create uncertainties that severely limit our ability to better understand the occurrence and magnitude of VE and to make confident estimations of the contribution of VE in the global terrestrial carbon balance. Based on these uncertainties, the algorithm was designed with restrictive assumptions that may result in the underestimation of the total impact of CO_2_ emissions released by VE, but that helped us test whether ventilation episodes occur globally (i.e., the main objective of our study). Furthermore, the incipient state of knowledge of this phenomenon hampers its detection when other processes simultaneously contribute to the net CO_2_ flux observed by the EC system. Future research should involve a better characterization of belowground CO_2_ production and transport processes by tracking the CO_2_ concentration and isotopic signal within the vadose zone of ecosystems where VE are likely to happen. The improvement of partitioning models would also reduce uncertainties in estimated biological CO_2_ exchanges. Nevertheless, the results achieved in this work are very relevant because the presence of VE has been identified in many arid and semiarid lands, which constitute the largest biome in the world (Schimel, 2010).

## 5 CONCLUSIONS

Due to the wide presence of arid and semiarid ecosystems, drylands' C balance strongly affects the inter‐annual variability of C dynamics at a global scale. In this sense, a more detailed information of drylands behavior is necessary to predict how climate change will affect their ecological structure and functions. The present study is a step toward a better understanding of the processes involved in the global C cycle. After analyzing eddy‐covariance open data observations of C fluxes and ancillary data from 145 common semiarid ecosystem types from FLUXNET and its main regional networks, we can confirm that subterranean ventilation CO_2_ emissions occur globally.

The occurrence and magnitude of CO_2_ emissions from the vadose zone towards the atmosphere were directly related to changes in friction velocity (indicator of atmospheric turbulence), the triggering variable. The soil water content and annual rainfall were found to be limiting factors. Only when the soil water content declines to values below the 30 percentile of site‐specific annual soil moisture, could the interconnectivity between vadose zone and the atmosphere allow for an effective gas transport across the land surface. These subterranean ventilated CO_2_ emissions occurred in dry ecosystems, but also in ecosystems where dryness was not an inherent feature of the ecosystem. Subterranean ventilation episodes varied from days to weeks, and occurred only during daytime. The Spearman coefficients obtained from partial correlation between u_*_ and CO_2_ fluxes were found to be similar (with a median value around 0.5) across the different climatic zones analyzed. Finally, the CO_2_ stored belowground available to be ventilated directly affects the occurrence and magnitude of CO_2_ emissions induced by VE.

Our results suggest that our algorithm can efficiently detect CO_2_ emissions produced by VE and constitutes a useful tool to its detection, for use in future research. Global warming and anthropogenic disturbance will increase the rate of drying in ecosystems leading to more frequent and intense drought events. This drying could provoke an ecosystem degradation favoring a greater exposure of subsoil CO_2_ to ventilation and affecting negatively in the trend of global carbon sequestration. Improving our knowledge of the carbon exchanges between terrestrial ecosystems and the atmosphere is essential to better understand and model the Earth's climate system. Understanding the occurrence of processes, feedbacks, and driving factors that modulate the carbon (C) source capacity of natural ecosystems due to ventilation episodes is needed to advance towards more robust model projections for future climate in order to predict how climate change will affect biology structure and functions as well as more adequate design of mitigation policies.

## CONFLICT OF INTEREST

The authors declare no competing financial interests.

## Supporting information


Table S1

Table S2

Table S3

## Data Availability

The algorithm's Matlab code is available at https://figshare.com/s/1b67062a4cc4b880b9a7. Data from experimental sites are freely available at FLUXNET2015 (https://fluxnet.fluxdata.org), AmeriFlux (http://ameriflux.lbl.gov), OzFlux (http://www.ozflux.org.au), and AsiaFlux (http://www.asiaflux.net) websites. Other data can be obtained by contacting the corresponding author.
