# Supplementary material for: Ecosystem CO2 release driven by wind occurs in drylands at global scale
Source: Glob Chang Biol. 2022 Jun 21;28(17):5320–33. doi: 10.1111/gcb.16277 (PMC9545467; doi:10.1111/gcb.16277)
Supplement: Supplementary file 1 — Table S1 [file GCB-28-5320-s002.docx]

**Table S1. Sites with ventilation episodes (VE). The table provides information for each ID site about (in order of appearance) the year analyzed; the five-day periods (beginning and ending day of years) when our algorithm detected VE-driven CO_2_ emissions according to the established criteria; the average of the maximum CO_2_ fluxes (F_c_) measured over all VE detected (F_c_ potential; µmol CO_2_ m^-2^ s^-1^); the mean and standard deviation values of Spearman coefficients obtained from partial correlation between F_c_ and friction velocity (u_*_) over all VE detected (F_c_-u_*_ corr; p < 0.05); the percentage of gaps in the F_c_ time series of one year duration (% gaps); and the sites citation information.**

| **Site ID** | **Year** | **Periods detected** | **F_c_ potential** | **F_c_-u_*_ corr** | **% gaps** | **Citation** |
| --- | --- | --- | --- | --- | --- | --- |
| AU-DaP | 2012 | 155-164, 170-189, 195-199, 210-224, 230-234, 240-269, 275-279, 285-294, 300-304 | 3.25 ± 0.69 | 0.57 ± 0.13 | 65 | Jason Beringer, Lindsay Hutley (2007-2013) FLUXNET2015 AU-DaP Daly River Savanna, DOI: 10.18140/FLX/1440123 |
| AU-Emr | 2013 | 260-264,275-279,285-289 | 3.21 ± 0.69 | 0.58 ± 0.14 | 53 | Ivan Schroder, Steve Zegelin, Tehani Palu, Andrew Feitz (2011-2013) FLUXNET2015 AU-Emr Emerald, DOI: 10.18140/FLX/1440198 |
| AU-RDF | 2013 | 135-139, 160-169 | 2.45 ± 0.57 | 0.40 ± 0.08* | 80 | Jason Beringer,  Lindsay Hutley  (2011-2013) FLUXNET2015 AU-RDF Red Dirt Melon Farm, Northern Territory, DOI: 10.18140/FLX/1440201 |
| BE-Lon | 2014 | 10-14, 25-34, 50-54, 60-64, 285-289 | 2.34 ± 1.03 | 0.49 ± 0.06 | 66 | Anne De Ligne,  Tanguy Manise,  Christine Moureaux,  Marc Aubinet, Bernard Heinesch  (2004-2014) FLUXNET2015 BE-Lon Lonzee, DOI: 10.18140/FLX/1440129 |
| CN-HaM | 2002 | 45-49, 55-59 | 3.66 ± 0.90 | 0.47 ± 0.08* | 44 | Yanhong Tang,  Tomomichi Kato,  Mingyuan Du  (2002-2004) FLUXNET2015 CN-HaM Haibei Alpine Tibet site, DOI: 10.18140/FLX/1440190 |
| ES-Amo | 2009 | 100-104,150-159,165-194, 200-249,335-344 | 4.86 ± 1.63 | 0.52 ± 0.15 | 37 | Francisco Domingo Poveda,  Ana López Ballesteros,  Erique Pérez Sánchez Cañete,  Penélope Serrano Ortiz,  Mª Rosario Moya Jiménez, Oscar Pérez Priego,  Andrew S. Kowalski  (2007-2012) FLUXNET2015 ES-Amo Amoladeras, DOI: 10.18140/FLX/1440156 |
| ES-Lju | 2009 | 170-179,205-209,235-244 | 2.61 ± 1.92 | 0.46 ± 0.16 | 48 | Erique Pérez Sánchez Cañete,  Penélope Serrano Ortiz,  Mª Rosario Moya Jiménez,  Francisco Domingo Poveda,  Oscar Pérez Priego,  Ana López Ballesteros,  Andrew S. Kowalski  (2004-2013) FLUXNET2015 ES-LJu Llano de los Juanes, 10.18140/FLX/1440157 |
| ES-Ln2 | 2009 | 150-154,165-199,205-209,215-219,230-249,265-269 | 4.52 ± 1.03 | 0.43 ± 0.16 | 66 | Andrew Kowalsky  (2009-2009) FLUXNET2015 ES-Ln2 Lanjaron-Salvage logging, DOI: 10.18140/FLX/1440226 |
| FR-Gri | 2006 | 265-279 | 6.46 ± 1.89 | 0.44 ± 0.07 | 63 | Pauline Buysse,  Brigitte Durand,  Jean-Christophe Gueudet,  Nicolas Mascher,  Eric Larmanou,  Pierre Cellier,  Benjamin Loubet  (2004-2014) FLUXNET2015 FR-Gri Grignon, 10.18140/FLX/1440162 |
| IT-Bci | 2008 | 260-269 | 6.52 ± 1.51 | 0.44 ± 0.07 | 67 | Vincenzo Magliulo,  Paul Di Tommasi,  Daniela Famulari,  Daniele Gasbarra,  Luca Vitale,  Antonio Manco  (2004-2014) FLUXNET2015 IT-BCi Borgo Cioffi, DOI: 10.18140/FLX/1440166 |
| RU-Cok | 2003 | 140-144, 150-154 | 1.38 ± 0.08 | 0.42 ± 0.21* | 78 | Han Dolman,  Michiel van der Molen,  Frans-Jan Parmentier,  Luca Belelli Marchesini,  Joshua Dean,  Ko van Huissteden,  Trofim Maximov  (2003-2014) FLUXNET2015 RU-Cok Chokurdakh, DOI: 10.18140/FLX/1440182 |
| RU-Ha1 | 2004 | 275-280,285-289 | 1.72 ± 0.22 | 0.44 ± 0.05* | 66 | Luca Belelli,  Dario Papale,  Riccardo Valentini  (2002-2004) FLUXNET2015 RU-Ha1 Hakasia steppe, DOI: 10.18140/FLX/1440184 |
| US-AR1 | 2011 | 50-54, 75-79,180-184,220-224, 235-239, 320-324 | 3.50 ± 1.82 | 0.37 ± 0.13 | 39 | Dave Billesbach,  James Bradford,  Margaret Torn  (2009-2012) FLUXNET2015 US-AR1 ARM USDA UNL OSU Woodward Switchgrass 1, DOI: 10.18140/FLX/1440103 |
| US-AR2 | 2010 | 200-204,220-224,240-244,260-264 | 3.38 ± 1.07 | 0.30 ± 0.06 | 35 | Dave Billesbach,  James Bradford,  Margaret Torn  (2009-2012) FLUXNET2015 US-AR2 ARM USDA UNL OSU Woodward Switchgrass 2, DOI: 10.18140/FLX/1440104 |
| US-ARM | 2003 | 155-159,160-164, 230-234 | 5.55 ± 0.59 | 0.41 ± 0.09 | 57 | Sebastien Biraud  (2003-2012) FLUXNET2015 US-ARM ARM Southern Great Plains site- Lamont, DOI: 10.18140/FLX/1440066 |
| US-IB2 | 2008 | 95-99,320-324,355-359 | 1.62 ± 0.46 | 0.45 ± 0.04* | 41 | Roser Matamala  (2004-2011) FLUXNET2015 US-IB2 Fermi National Accelerator Laboratory- Batavia (Prairie site), DOI: 10.18140/FLX/1440072 |
| US-LWW | 1998 | 95-99, 190-199,205-214,220-224, 230-234 | 8.07 ± 2.47 | 0.47 ± 0.15 | 53 | Tilden Meyers  (1997-1998) FLUXNET2015 US-LWW Little Washita Watershed, DOI: 10.18140/FLX/1440077 |
| US-Ne1 | 2002 | 95-104 | 3.33 ± 1.03 | 0.46 ± 0.13 | 47 | Andy Suyker  (2001-2013) FLUXNET2015 US-Ne1 Mead - irrigated continuous maize site, 10.18140/FLX/1440084 |
| US-Ne2 | 2008 | 100-104, 280-284 | 3.73 ± 0.43 | 0.49 ± 0.18 | 41 | Andy Suyker  (2001-2013) FLUXNET2015 US-Ne2 Mead - irrigated maize-soybean rotation site, DOI: 10.18140/FLX/1440085 |
| US-Ne3 | 2004 | 290-304 | 5.56 ± 3.24 | 0.52 ± 0.11 | 44 | Andy Suyker  (2001-2013) FLUXNET2015 US-Ne3 Mead - rainfed maize-soybean rotation site, 10.18140/FLX/1440086 |
| US-SRG | 2011 | 160-164, 350-354 | 2.37 ± 1.17 | 0.55 ± 0.08* | 40 | Russell Scott (2008-2014) FLUXNET2015 US-SRG Santa Rita Grassland, DOI: 10.18140/FLX/1440114 |
| US-Twt | 2011 | 65-69, 160-164 | 3.77 ± 2.30 | 0.32 ± 0.12* | 67 | Dennis Baldocchi  (2009-2014) FLUXNET2015 US-Twt Twitchell Island, DOI: 10.18140/FLX/1440106 |
| US-Var | 2013 | 160-164,180-184, 240-244,275-279 | 3.64 ± 1.05 | 0.27 ± 0.02 | 55 | Dennis Baldocchi  (2000-2014) FLUXNET2015 US-Var Vaira Ranch- Ione, DOI: 10.18140/FLX/1440094 |
| US-Whs | 2014 | 40-44,145-149,165-169 | 1.53 ± 0.38 | 0.34 ± 0.13 | 52 | Russ Scott  (2007-2014) FLUXNET2015 US-Whs Walnut Gulch Lucky Hills Shrub, DOI: 10.18140/FLX/1440097 |
| US-Wkg | 2011 | 5-9, 55-59,65-69,310-314,350-354 | 1.50 ± 0.29 | 0.49 ± 0.13 | 62 | Russell Scott  (2004-2014) FLUXNET2015 US-Wkg Walnut Gulch Kendall Grasslands, DOI: 10.18140/FLX/1440096 |
| ZA-Kru | 2007 | 65-69,75-79,125-129,155-159,160-164,170-179,190-194,200-204,270-274,330-334 | 7.36 ± 1.39 | 0.44 ± 0.08 | 56 | Bob Scholes  (2000-2013) FLUXNET2015 ZA-Kru Skukuza, 10.18140/FLX/1440188 |
| MX-Lpa | 2006 | 40-44, 105-114, 180-184, 190-194,325-329 | 9.90 ± 6.77 | 0.37 ± 0.14 | 2 | Walter Oechel AmeriFlux MX-Lpa La Paz, DOI:10.17190/AMF/1246014 |
| US-A74 | 2016 | 35-39,90-94,95-99,315-319 | 6.74 ± 1.97 | 0.53 ± 0.18 | 43 | Lara Kueppers, Margaret Torn, Sebastien Biraud AmeriFlux US-A74 ARM SGP milo field, DOI:10.17190/AMF/1436328 |
| US-Aud | 2005 | 15-19, 30-34, 45-49, 55-64, 95-99, 115-119, 125-129, 135-139, 150-174, 180-194, 330-334 | 4.89 ± 1.21 | 0.49 ± 0.17 | 12 | Tilden Meyers AmeriFlux US-Aud Audubon Research Ranch, DOI:10.17190/AMF/1246028 |
| US-Bo1 | 2006 | 55-59, 265-269, 300-304 | 7.13 ± 4.30 | 0.53 ± 0.08 | 23 | Tilden Meyers AmeriFlux US-Bo1 Bondville, DOI:10.17190/AMF/1246036 |
| US-Bo2 | 2007 | 80-84, 130-134 | 4.74 ± 1.62 | 0.37 ± 0.06* | 74 | Carl Bernacchi AmeriFlux US-Bo2 Bondville (companion site), DOI:10.17190/AMF/1246037 |
| US-Br1 | 2009 | 305-314 | 1.60 ± 0.27 | 0.29 ± 0.06* | 3 | John Prueger, Tim Parkin AmeriFlux US-Br1 Brooks Field Site 10- Ames, DOI:10.17190/AMF/1246038 |
| US-Ctn | 2007 | 180-184,210-214 | 7.13 ± 0.56 | 0.55 ± 0.06* | 23 | Tilden Meyers AmeriFlux US-Ctn Cottonwood, DOI:10.17190/AMF/1246117 |
| US-Fwf | 2009 | 310-314, 320-324 | 0.55 ± 0.17 | 0.47 ± 0.01* | 2 | Sabina Dore, Thomas Kolb AmeriFlux US-Fwf Flagstaff - Wildfire, DOIi:10.17190/AMF/1246052 |
| US-IB1 | 2007 | 75-79,110-114,135-139 | 4.23 ± 1.47 | 0.37 ± 0.13 | 18 | Roser Matamala AmeriFlux US-IB1 Fermi National Accelerator Laboratory- Batavia (Agricultural site), DOI:10.17190/AMF/1246065 |
| US-Kon | 2010 | 100-104, 335-339 | 3.86 ± 2.67 | 0.34 ± 0.01* | 33 | Nathaniel Brunsell AmeriFlux US-Kon Konza Prairie LTER (KNZ), DOI:10.17190/AMF/1246068 |
| US-Rms | 2015 | 200-204,185-189 | 2.68 ± 0.32 | 0.51 ± 0.16 | 28 | Gerald Flerchinger AmeriFlux US-Rms RCEW Mountain Big Sagebrush, DOI:10.17190/AMF/1375202 |
| US-SCd | 2011 | 110-114, 140-144, 155-159, 180-184, 270-274, 335-339 | 3.55 ± 3.01 | 0.51 ± 0.16 | 7 | Mike Goulden AmeriFlux US-SCd Southern California Climate Gradient - Sonoran Desert, DOI:10.17190/AMF/1419505 |
| US-SCg | 2014 | 145-149,155-159,165-179, 185-194, 220-224 | 6.15 ± 2.34 | 0.44 ± 0.10 | 32 | Mike Goulden AmeriFlux US-SCg Southern California Climate Gradient - Grassland, DOI:10.17190/AMF/1419502 |
| US-SCw | 2012 | 130-134, 140-149, 155-159, 165-169, 180-189, 200-209, 220-224, 245-249,260-264,295-299,305-309, 320-324 | 4.01 ± 1.36 | 0.52 ± 0.17 | 3 | Mike Goulden AmeriFlux US-SCw Southern California Climate Gradient - Pinyon/Juniper Woodland, doi:10.17190/AMF/1419504 |
| US-Seg | 2011 | 55-59,140-144,155-159,335-339,355-359 | 1.55 ± 0.54 | 0.33 ± 0.07 | 27 | Marcy Litvak AmeriFlux US-Seg Sevilleta grassland, DOI:10.17190/AMF/1246124 |
| US-SFP | 2008 | 100-104,280-284 | 4.13 ± 1.65 | 0.33 ± 0.03* | 39 | Tilden Meyers AmeriFlux US-SFP Sioux Falls Portable, DOI:10.17190/AMF/1246126 |
| US-SO4 | 2006 | 35-39, 225-229, 305-309, 320-324, 335-339 | 4.15 ± 2.87 | 0.55 ± 0.13 | 17 | Walt Oechel AmeriFlux US-SO4 Sky Oaks- New Stand, DOI:10.17190/AMF/1246099 |
| US-SRM | 2006 | 5-14,35-44,135-144,165-174,200-204 | 5.72 ± 5.89 | 0.37 ± 0.14 | 10 | Marcy Litvak AmeriFlux US-Wjs Willard Juniper Savannah, DOI:10.17190/AMF/1246120 |
| US-Wjs | 2013 | 20-24,45-49,60-64,70-74, 80-84,100-104,120-124,135-149, 140-144, 145-149, 170-174 | 2.21 ± 0.65 | 0.40 ± 0.10 | 18 | Marcy Litvak AmeriFlux US-Wjs Willard Juniper Savannah, DOI:10.17190/AMF/1246120 |
| CN-HBG | 2003 | 10-15, 20-25, 30-49, 290-294, 300-304, 345-349 | 2.92 ± 1.57 | 0.55 ± 0.12 | 9 | Xinquan Zhao, Asiaflux, CN-QHB, Haibei Potentilla fruticisa bosk Site |
| CN-QHB | 2002 | 45-49, 55-59, 310-314 | 4.49 ± 1.35 | 0.40 ± 0.13 | 59 | Yanhong Tang, AsiaFlux, CN-QHB, Qinghai Flux Research Site |
| AU-Stp | 2010 | 155-164, 185-199, 225-234, 250-254, 270-274 | 1.45 ± 0.54 | 0.38 ± 0.12 | 16 | Jason Beringer,  Lindsay Hutley  (2008-2014) FLUXNET2015 AU-Stp Sturt Plains, DOI: 10.18140/FLX/1440204 |
| AU-TTE | 2013 | 5-9 15-34, 45-49, 70-74, 80-84, 120-129, 180-184, 200-204, 210-214, 240-244, 255-269, 280-289, 295-299 | 3.14 ± 1.32 | 0.55 ± 0.16 | 23 | James Cleverly,  Derek Eamus  (2012-2014) FLUXNET2015 AU-TTE Ti Tree East, DOI: 10.18140/FLX/1440205 |
| AU-Ync | 2014 | 290-294, 300-304, 340-344, 360-364 | 5.43 ± 2.36 | 0.52 ± 0.07 | 16 | Jason Beringer,  Jeffery Walker  (2012-2014) FLUXNET2015 AU-Ync Jaxa, DOI: 10.18140/FLX/1440208 |
