# Supplementary material for: Ecosystem CO2 release driven by wind occurs in drylands at global scale
Source: Glob Chang Biol. 2022 Jun 21;28(17):5320–33. doi: 10.1111/gcb.16277 (PMC9545467; doi:10.1111/gcb.16277)
Supplement: Supplementary file 2 — Table S2 [file GCB-28-5320-s003.docx]

**Table S2. Sites with ventilation episodes. The table provides information about the main characteristics of experimental sites with information for each ID site about (in order of appearance) land cover IGBP classification (IGBP), Köppen climate classification (KGCC), latitude, longitude, mean annual temperature (MAT; °C); mean annual precipitation (MAP; mm), network to which the database belongs and associated DOI.**

| **Site ID** | **Site Country** | **IGBP** | **KGCC** | **Latitude** | **Longitude** | **MAT** | **MAP** | **Data Base** | **DOI** |
| --- | --- | --- | --- | --- | --- | --- | --- | --- | --- |
| AU-DaP | Australia | GRA | Aw | -14.159 | 131.388 | 25 | 801 | Fluxnet | <http://dx.doi.org/10.18140/FLX/1440123> |
| AU-Emr | Australia | GRA | BWk | -23.859 | 148.475 | 22 | 527 | Fluxnet | <http://dx.doi.org/10.18140/FLX/1440198> |
| AU-RDF | Australia | WSA | BWh | -14.564 | 132.478 | 26 | 600 | Fluxnet | <http://dx.doi.org/10.18140/FLX/1440201> |
| BE-Lon | Belgium | CRO | Cfb | 50.552 | 4.746 | 10 | 800 | Fluxnet | <http://dx.doi.org/10.18140/FLX/1440129> |
| CN-HaM | China | GRA | ET | 37.370 | 101.180 | -1 | 565 | Fluxnet | <http://dx.doi.org/10.18140/FLX/1440190> |
| ES-Amo | Spain | OSH | BSk | 36.834 | -2.252 | 18 | 220 | Fluxnet | <http://dx.doi.org/10.18140/FLX/1440156> |
| ES-Lju | Spain | OSH | Csa | 36.927 | -2.752 | 16 | 400 | Fluxnet | <http://dx.doi.org/10.18140/FLX/1440157> |
| ES-Ln2 | Spain | OSH | Csa | 36.970 | -3.476 | 6 | 800 | Fluxnet | <http://dx.doi.org/10.18140/FLX/1440226> |
| FR-Gri | France | CRO | Cfb | 48.844 | 1.952 | 12 | 650 | Fluxnet | <http://dx.doi.org/10.18140/FLX/1440162> |
| IT-Bci | Italy | CRO | Csa | 40.524 | 14.957 | 18 | 600 | Fluxnet | <http://dx.doi.org/10.18140/FLX/1440166> |
| RU-Cok | Russia | OSH | Dfc | 70.829 | 147.494 | -14 | 232 | Fluxnet | <http://dx.doi.org/10.18140/FLX/1440182> |
| RU-Ha1 | Russia | GRA | Dfc | 54.725 | 90.002 | 2 | 384 | Fluxnet | <http://dx.doi.org/10.18140/FLX/1440184> |
| US-AR1 | United States | GRA | Cfa | 36.427 | -99.420 | 14 | 550 | Fluxnet | <http://dx.doi.org/10.18140/FLX/1440103> |
| US-AR2 | United States | GRA | Cfa | 39.636 | -99.598 | 15 | 419 | Fluxnet | <http://dx.doi.org/10.18140/FLX/1440104> |
| US-ARM | United States | CRO | Cfa | 36.606 | -97.489 | 15 | 843 | Fluxnet | <http://dx.doi.org/10.18140/FLX/1440066> |
| US-IB2 | United States | GRA | Dfa | 41.841 | -88.241 | 9 | 930 | Fluxnet | <http://dx.doi.org/10.18140/FLX/1440072> |
| US-LWW | United States | GRA | Cfa | 34.960 | -97.979 | 16 | 805 | Fluxnet | <http://dx.doi.org/10.18140/FLX/1440077> |
| US-Ne1 | United States | CRO | Dfa | 41.165 | -96.477 | 10 | 790 | Fluxnet | <http://dx.doi.org/10.18140/FLX/1440084> |
| US-Ne2 | United States | CRO | Dfa | 41.165 | *-96.470* | 10 | 789 | Fluxnet | <http://dx.doi.org/10.18140/FLX/1440085> |
| US-Ne3 | United States | CRO | Dfa | 41.180 | -96.439 | 10 | 784 | Fluxnet | <http://dx.doi.org/10.18140/FLX/1440086> |
| US-SRG | United States | GRA | BSk | 31.908 | -110.839 | 17 | 420 | Fluxnet | <http://dx.doi.org/10.18140/FLX/1440114> |
| US-Twt | United States | CRO | Csa | 38.109 | -121.653 | 16 | 421 | Fluxnet | <http://dx.doi.org/10.18140/FLX/1440106> |
| US-Var | United States | GRA | Csa | 38.413 | -120.951 | 16 | 559 | Fluxnet | <http://dx.doi.org/10.18140/FLX/1440094> |
| US-Whs | United States | OSH | BSk | 31.744 | -110.052 | 18 | 320 | Fluxnet | <http://dx.doi.org/10.18140/FLX/1440097> |
| US-Wkg | United States | GRA | BSk | 31.737 | -109.942 | 16 | 407 | Fluxnet | <http://dx.doi.org/10.18140/FLX/1440096> |
| ZA-Kru | South Africa | SAV | BSh | -25.020 | 31.497 | 22 | 547 | Fluxnet | <http://dx.doi.org/10.18140/FLX/1440188> |
| MX-Lpa | Mexico | OSH | BWh | 24.129 | -110.438 | 24 | 182 | AmeriFlux | <http://dx.doi.org/10.17190/AMF/1246014> |
| US-A74 | United States | CRO | Cfa | 36.809 | -97.549 | 34 | 889 | AmeriFlux | <http://dx.doi.org/10.17190/AMF/1436328> |
| US-Aud | United States | GRA | BSk | 31.591 | -110.510 | 15 | 438 | AmeriFlux | <http://dx.doi.org/10.17190/AMF/1246028> |
| US-Bo1 | United States | CRO | Dfa | 40.006 | -88.290 | 11 | 991 | AmeriFlux | <http://dx.doi.org/10.17190/AMF/1246036> |
| US-Bo2 | United States | CRO | Dfa | 40.009 | -88.290 | 11 | 991 | AmeriFlux | <http://dx.doi.org/10.17190/AMF/1246037> |
| US-Br1 | United States | CRO | Dfa | 41.975 | -93.691 | 9 | 842 | AmeriFlux | <http://dx.doi.org/10.17190/AMF/1246038> |
| US-Ctn | United States | GRA | BSk | 43.950 | -101.847 | 10 | 228 | AmeriFlux | <http://dx.doi.org/10.17190/AMF/1246117> |
| US-Fwf | United States | GRA | Csb | 35.445 | -111.772 | 8 | 557 | AmeriFlux | <http://dx.doi.org/10.17190/AMF/1246052> |
| US-IB1 | United States | CRO | Dfa | 41.859 | -88.241 | 9 | 929 | AmeriFlux | <http://dx.doi.org/10.17190/AMF/1246065> |
| US-Kon | United States | GRA | Cfa | 39.082 | -96.560 | 13 | 867 | AmeriFlux | <http://dx.doi.org/10.17190/AMF/1246068> |
| US-Rms | United States | CSH | BSh | 43.065 | -116.749 | 5 | 800 | AmeriFlux | <http://dx.doi.org/10.17190/AMF/1375202> |
| US-SCd | United States | BSV | BWh | 33.652 | -116.372 | 23 | 350 | AmeriFlux | <http://dx.doi.org/10.17190/AMF/1419505> |
| US-SCg | United States | GRA | Csa | 33.737 | -117.695 | 17 | 350 | AmeriFlux | <http://dx.doi.org/10.17190/AMF/1419502> |
| US-SCw | United States | OSH | BWh | 33.605 | -116.453 | 17 | 350 | AmeriFlux | <http://dx.doi.org/10.17190/AMF/1419504> |
| US-Seg | United States | GRA | BSk | 34.362 | -106.702 | 14 | 273 | AmeriFlux | <http://dx.doi.org/10.17190/AMF/1246124> |
| US-SFP | United States | CRO | Csa | 43.241 | -96.902 | 8 | 494 | AmeriFlux | <http://dx.doi.org/10.17190/AMF/1246126> |
| US-SO4 | United States | CSH | Csa | 33.385 | -116.641 | 15 | 484 | AmeriFlux | <http://dx.doi.org/10.17190/AMF/1246099> |
| US-SRM | United States | WSA | BSk | 31.821 | -110.866 | 18 | 380 | AmeriFlux | <http://dx.doi.org/10.17190/AMF/1246120> |
| US-Wjs | United States | OSH | BSk | 34.426 | -105.862 | 15 | 361 | AmeriFlux | <http://dx.doi.org/10.17190/AMF/1246120> |
| CN-HBG | China | GRA | Dwb | 37.482 | 101.200 | -2 | 600 | AsiaFlux | <http://asiaflux.net/?page_id=1176> |
| CN-QHB | China | GRA | BSk | 37.607 | 101.332 | -2 | 567 | AsiaFlux | <http://asiaflux.net/index.php?page_id=94> |
| AU-Stp | Australia | GRA | BSh | -17.151 | 133.350 | 25 | 640 | OzFlux | <http://dx.doi.org/10.18140/FLX/1440204> |
| AU-TTE | Australia | OSH | BWh | -22.287 | 133.640 | 21 | 305 | OzFlux | <http://dx.doi.org/10.18140/FLX/1440205> |
